# Temporal stability and assignment power of adaptively divergent genomic regions between herring (*Clupea harengus*) seasonal spawning aggregations

**DOI:** 10.1002/ece3.4768

**Published:** 2018-12-11

**Authors:** Quentin Kerr, Angela P. Fuentes‐Pardo, James Kho, Jenni L. McDermid, Daniel E. Ruzzante

**Affiliations:** ^1^ Department of Biology Dalhousie University Halifax Nova Scotia Canada; ^2^ Marine Fish and Mammals Section, Fisheries and Oceans Canada Gulf Fisheries Centre Moncton New Brunswick Canada

**Keywords:** adaptive divergence, fisheries, management, population genomics, SNP panel, temporal stability

## Abstract

Atlantic herring (*Clupea harengus*), a vital ecosystem component and target of the largest Northwest Atlantic pelagic fishery, undergo seasonal spawning migrations that result in elusive sympatric population structure. Herring spawn mostly in fall or spring, and genomic differentiation was recently detected between these groups. Here we used a subset of this differentiation, 66 single nucleotide polymorphisms (SNPs) to analyze the temporal dynamics of this local adaptation and the applicability of SNP subsets in stock assessment. We showed remarkable temporal stability of genomic differentiation corresponding to spawning season, between samples taken a decade apart (2005 *N* = 90 vs. 2014 *N* = 71) in the Gulf of St. Lawrence, and new evidence of limited interbreeding between spawning components. We also examined an understudied and overexploited herring population in Bras d'Or lake (*N* = 97); using highly reduced SNP panels (*N*_SNPs_ > 6), we verified little‐known sympatric spawning populations within this unique inland sea. These results describe consistent local adaptation, arising from asynchronous reproduction in a migratory and dynamic marine species. Our research demonstrates the efficiency and precision of SNP‐based assessments of sympatric subpopulations; and indeed, this temporally stable local adaptation underlines the importance of such fine‐scale management practices.

## 1 INTRODUCTION

Highly abundant and widely distributed marine fish species generally exhibit large effective population sizes, a condition that enhances the efficiency of natural selection while minimizing genetic drift (Fraser et al., 2007; Gossmann, Keightley, & Eyre‐Walker, 2012). Historically, identification of population structure in marine species has relied on variability at neutral markers, capturing differentiation resulting from genetic drift (Luck, Daily, & Ehrlich, 2003). Perhaps unsurprisingly, past attempts to identify fine‐scale population structure in abundant marine species using such markers have either not identified structure (André et al., 2011; Carr, Snellen, Howse, & Wroblewski, 1995; Dahle, 1991; Limborg et al., 2012; Westgaard & Fevolden, 2007) or if detected, structure was apparent in a minority of loci often subsequently found to be linked to functional genes (e.g., genes involved in salinity adaptation [André et al., 2011; Bekkevold et al.., 2005; Gaggiotti et al., 2009])*.* Recently, adaptive SNP‐based approaches have shown increased power over neutral markers regarding population structuring (Ackerman, Habicht, & Seeb, 2011; André et al., 2011; Limborg et al., 2012; Nielsen et al., 2012; Speller et al., 2012). Adaptive variation may, however, shift due to fluctuating selective pressures, with little repeatability over time and hence little predictive value (Poulsen, Hemmer‐Hansen, Loeschcke, Carvalho, & Nielsen, 2011; Therkildsen, Hemmer‐Hansen, Als, et al., 2013; Therkildsen, Hemmer‐Hansen, Hedeholm, et al., 2013).

Atlantic herring (*Clupea harengus*) is an ideal species for studying spatial and temporal population structuring and local adaptation in the sea. It is one of the most abundant and widely distributed fish in the Northwest Atlantic (DFO, 2017), reaches maturity at the age of 3 to 4 years, and exhibits an intricate life history involving migrations among overwintering, feeding, and spawning aggregations. These characteristics make the identification of population structure challenging (Iles & Sinclair, 1982). Certainly, the species’ extensive dispersal capabilities (McQuinn, 1997a) suggests slow rates of population differentiation (Kawecki & Ebert, 2004). Reproductive strategies in Atlantic herring have been well studied and are the basis of distinguishable subpopulations. Spawning occurs in multiple spawning events at predictable locations, discrete in space and time; past tagging studies have suggested that herring exhibit strong site fidelity (Stephenson, Melvin, & Power, 2009; Wheeler & Winters, 1984). In many parts of the Northwest Atlantic, herring spawn in both spring and fall, resulting in sympatric components that frequently overlap outside the reproductive season (Stephenson et al., 2009). Seasonal spawning components exhibit different growth rates, abundance trends (Harma, Brophy, Minto, & Clarke, 2012; Melvin, Stephenson, & Power, 2009), and morphologies (Messieh, 1975), and are managed separately in the Gulf of St. Lawrence (GSL) (DFO, 2016a). Shifts in the relative abundance of these two components have recently occurred in Atlantic Canada: spring spawning herring have declined substantially in the GSL (DFO, 2016a) and off Newfoundland (DFO, 2016b), and almost entirely disappeared from the coast of Quebec (DFO, 2002). The reasons for such shifts are largely unknown but may be aggravated by changing temperatures (Arula, Raid, Simm, & Ojaveer, 2016; Melvin et al., 2009) or seasonally shifting fishing pressure (Jardine & Sanchirico, 2015).

Recently, over 6,000 SNPs were shown to exhibit significant differentiation between spring and fall spawning aggregations (Lamichhaney et al., 2017). Much of this divergence was in the vicinity of genes, some that have an established role in reproduction (Lamichhaney et al., 2017). Discriminant methods based on adaptive markers such as these have broad application for the monitoring of population diversity (Laikre et al., 2008); Thus, the optimization of SNP‐based characterization is of ongoing interest (Ding et al., 2011; Storer et al., 2012; Wilkinson et al., 2011).

Here, we analyze the patterns of genomic differentiation between spring and fall spawning components in the Northwest Atlantic. First, we examined the long‐term (~10 years) temporal stability of genomic differentiation between seasonal spawning components in the GSL and examined evidence for spawning‐component interbreeding. We screened 66 SNPs, representing 10 genomic scaffolds found by Lamichhaney et al. (2017) to distinguish between spring and fall spawning herring. This SNP panel consisted of 32 variants that differentiated between spawning components in the Northwest Atlantic (henceforth the Northwest‐only set) and 34 variants that differentiated between spawning components on both sides of the Atlantic Ocean (henceforth the Transatlantic set). Secondly, we explored the degree to which a smaller number of SNPs could be used for spawning season individual assignment: we created subsets of these same 66 SNPs, using marker ranking and thinning techniques. Finally, we used the different SNP panels to assess the reproductive components of an understudied and overexploited herring population from the Bras d'Or Lake (BDO).

## 2 METHODS

### 2.1 Sample collection

A total of 276 adult herring were collected during spring and fall spawning seasons from two locations in the GSL in 2005 and 2014, and in the spring season from BDO in 2016 and 2017 (Supporting Information Table S1). GSL individuals were used for two purposes. First, for the assessment of long‐term temporal stability of genetic differentiation between spring and fall spawning herring. Second, for designing an optimized and cost‐effective SNP panel for the genetic identification of spawning season in Atlantic herring. The complete and reduced SNP panels were further used for disentangling the spawning composition of herring from BDO. Individual gonadal maturation state was visually diagnosed by researchers from Fisheries and Oceans Canada (DFO) (2008). A muscle or fin sample was taken from each individual and stored in 95% ethanol at −20°C.

### 2.2 DNA extraction and SNP genotyping

Total genomic DNA was extracted following a standard phenol‐chloroform protocol. DNA quality was assessed in 0.8% agarose gel electrophoresis (0.5× TBE buffer) using a 1 Kb molecular weight ladder. DNA quantity was measured using the Quant‐iT PicoGreen dsDNA assay (Thermo Fisher Scientific) with a Roche LightCycler 480 Instrument (Roche Molecular Systems, Inc.). Given that DNA of archived samples may exhibit different levels of degradation, we evaluated whether these samples were suitable for the Polymerase Chain Reaction (PCR), and thus, for SNP genotyping. For this, in 8 randomly chosen individuals with fragmented DNA, we amplified by PCR a total of 66 SNP loci reported in Lamichhaney et al. (2017) as strongly associated with spawning time in Atlantic herring. In this SNP panel, 34 loci were informative in populations on both sides of the Atlantic, and 32 were exclusively discriminatory in the West Atlantic (Supporting Information Table S3). Master mix preparation for PCR amplification followed McCracken et al. (2014) and cycle protocol followed Gabriel, Ziaugra, and Tabbaa (2009) with some modification (Supporting Information Table S4). Amplification products were visualized in 1% agarose gel electrophoresis (0.5× TBE buffer) with a 100 bp molecular weight ladder. Based on the results of this pilot test, we submitted all 276 DNA samples to *Neogen Corporation* (Lincoln, U.S.) for genotyping in the 66 SNP panel using the Agena MassARRAY system.

### 2.3 Quality control of raw data and population structure analysis

Individuals or loci with more than 10% missing data were removed from further analysis via *PLINK *(Purcell et al., 2007). SNP genotypes were phased using BEAGLE (Ayres et al., 2012). To explore individual and population clustering patterns, we performed a Principal Component Analysis (PCA) using the R package *adegenet* (Jombart, 2008). To examine the distribution of genetic variation within and between years and locations, we performed a hierarchical analysis of molecular variance (AMOVA). This analysis was run on all loci as well as on a locus‐by‐locus basis, via *Arlequin* (Excoffier & Lischer, 2010). For both AMOVAs, collection year (*F*_SC_) was nested within spawning season (*F*_ST_). The proportion of heterozygosity (PHt) was calculated for all individuals using GENHET (Coulon, 2010), and the program NewHybrids (Lamichhaney et al., 2017) was used to assess the number and type of hybrids that may occur between the two spawning components; previous research suggests that these loci should be able to identify hybrid classes resulting from *n* = 2 generations of hybridization (Vähä & Primmer, 2006).

### 2.4 SNP panel optimization

Two steps were used to design optimal SNP panels with which to characterize the spawning season of herring. First, SNPs were ranked according to four metrics using the GSL samples (samples with gonadally validated spawning season): *WHICHLOCI* scores (WLS) (Banks, Eichert, & Olsen, 2003), *F*_ST_, *I*_N_, and *δ* (Kavakiotis et al., 2015). Using these ranking methods, the spawning season assignment accuracy of increasingly small SNP panels was tested. This was done by creating a discriminant analysis of principle components (DAPC) using 90% of the GSL spring and fall spawners, and then testing the accuracy of this model on the remaining 10% of the sample (via R package *adegenet* [Jombart, 2008]). This process was repeated 1,000 times per panel. Further thinning of SNPs was performed using the metric that resulted in the highest proportion of accuracy.

Secondly, three further SNP thinning methods were used, each matching a specific objective: (a) Panel of loci on separate scaffolds: only the top ranked SNP in each scaffold was chosen. (b) Panel of loci with low redundancy: To minimize redundant information, loci underwent complete linkage clustering, based on 1 – *R* ^2^. SNPs with *R* ^2^ > 0.5 were clustered, and only the top ranked SNP per cluster was used. (c) Panel of only transatlantic loci: To maximize geographic applicability, only SNPs that were found in both the Northwest and NE were used. These three SNP sets were then further reduced using the best ranking method as assessed above. Optimal panels (i.e., those attaining the highest accuracy with the least SNPs) were found for the three screened subsets of loci, as well as all 64 unscreened loci.

### 2.5 Analysis of BDO samples

BDO herring has been categorized as predominantly spring spawning, though fall spawning may occur (McPherson, 2001), and as such represent an interesting case study. We assessed the spawning composition in BDO using the whole SNP panel and the different subsets designed. These herring were sampled in the spring, but the majority were not actively spawning. As such, the spawning season of this set was almost entirely unknown; these samples were assigned to either fall or spring spawning components using DAPC based on the SNP sets designed above.

## 3 RESULTS

### 3.1 SNP genotyping and genetic structuring

**Figure 1:**
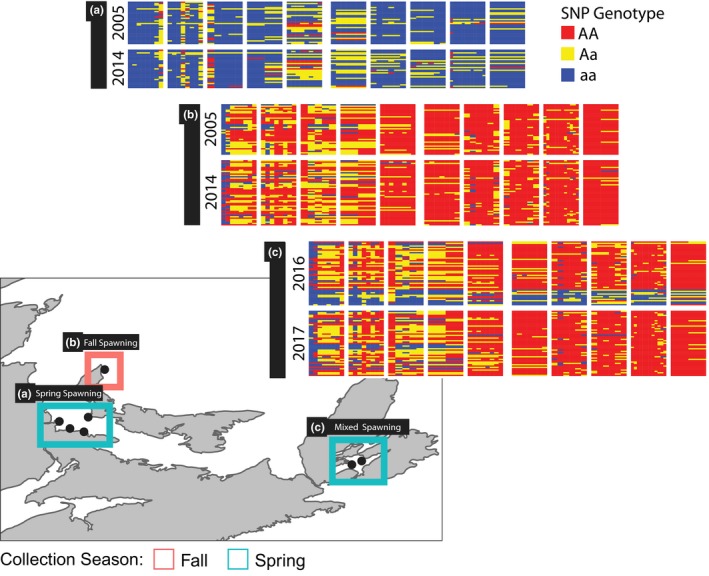
Genotypes of individuals (heat map rows) at 64 SNPs divided by genomic regions (heat map columns), and sample locations based on port of landing of the fishing vessel. Note that collection season corresponds to spawning season in the GSL (a and b), as only actively spawning herring were sampled; in BDO (c) fish were indiscriminately collected in the spring

A total of 276 Atlantic herring (30 of which were used in Lamichhaney et al., 2017 [Supporting Information Table S1]) were genotyped at 66 SNP loci previously shown to discriminate between spring and fall spawning herring. Two of the 66 loci failed in >10% of individuals and were removed from further analysis (Supporting Information Figure S1). Furthermore, 31 individuals failed at >10% of SNP sites and were also excluded (Supporting Information Figure S2). Thus, at least 58 SNPs were successfully genotyped in each of the remaining 245 individuals Figure 1). Of these, *N* = 148 were collected from the GSL (2005: *N* = 70, 2014: *N* = 78) while actively spawning in the spring (*N* = 61) or fall (*N* = 87). The remaining *N* = 97 individuals were collected from BDO in the spring but comprised both spawning (*N* = 12) and nonspawning individuals (*N* = 85) (Supporting Information Table S1).

**Figure 2:**
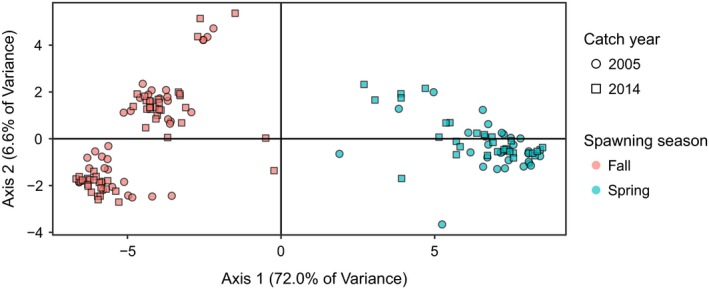
Principal Components Analysis plot reflecting the genetic composition of *N* = 148 herring from the GSL. The genetic information is based on 64 SNPs known to discriminate between spring and fall spawning individuals (Lamichhaney et al., 2017). Adult herring in the GSL cluster by spawning season along the first Principal Component which explains 72.0% of the total variance

**Figure 3:**
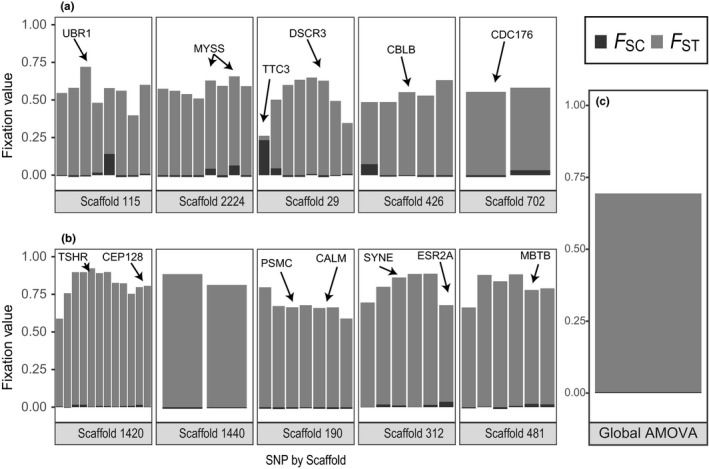
The AMOVA indices of 64 SNPs genotyped in *N* = 148 herring with known spawning season from the GSL: 31 individual SNPs unique to the Northwest Atlantic (a), 33 individual transatlantic SNPs (b), and all 64 combined SNPs (c). *F*_ST_ refers to differentiation between spawning season, samples, while *F*_SC_ refers to differentiation between sampling years. Genes within 5 kbp from the SNP are labeled following Lamichhaney et al. (2017)

A Principal Components Analysis (PCA) based on 64 SNPs clustered individuals by spawning season regardless of collection year (Figure 2). A global AMOVA showed that 69.32% of variation is explained by spawning season, while 0.08% of variation is explained by differences between 2005 and 2014 (Table 1). Accordingly, the global *F*_ST_ between spawning components was high (*F*_ST_ = 0.69, *p* < 0.0001, 1,023 iterations), while both *F*_SC_ and *F*_CT_ were insignificant (*p* > 0.25, 1,023 iterations) (Table 1). On a locus‐by‐locus basis, all 64 SNPs were highly divergent between spawning season components, both regarding the Northwest‐only SNP set (Figure 3a) and the transatlantic SNP set (Figure 3b). Although the loci from the transatlantic set were generally more divergent, all *F*_ST_ estimates were significant at *α* = 0.05, with a False Discovery Rate (FDR) correction (Supporting Information Table S2). Many of the highly significant *F*_ST_ estimates corresponded to SNP sites located within 5 kb of genes, as reported by Lamichhaney et al. (2017) (Figure 3). A small number of loci from the Northwest‐only SNP set appeared to show non‐negligible *F*_SC_ values, one of which was significant after FDR correction (Supporting Information Table S2), but generally divergence between collection years was negligible (Figure 3).

**Table 1:** Global AMOVA, conducted over all 64 loci with filtered samples from the Gulf of St. Lawrence—where spawning season was verified to match catch season

Source of variation	*df*	Sum of squares	Variance components	Percentage of variation	Fixation indices	*p*‐Value
Among spawning seasons	1	1802.41	11.77	69.32	*F*_ST_ = 0.694	<0.0001
Among years within spawning seasons	2	12.41	0.01	0.08	*F*_SC_ = 0.003	0.26 ± 0.01
Within years	306	1588.98	5.19	30.60	*F*_CT_ = 0.693	0.33 ± 0.02
Total	309	3,403.80	16.97			

Note

Samples from 2005 and 2014 were used (*N* = 148).

### 3.2 SNP ranking and SNP panel size reduction

All four SNP ranking metrics, WL (Banks et al., 2003), *F*_ST_, *I*_N_, and *δ* (Kavakiotis et al., 2015) were highly correlated; the top 27 SNPs (according to any method) could distinguish between fall and spring spawners with 100% cross‐validated accuracy, using a Discriminant Analysis of Principal Components (DAPC) (Supporting Information Figure S3). DAPC is an easily applicable method for minimizing within‐group differences while maximizing between‐group differences using principal components, which can be used to assign individuals to genetic subpopulations (Jombart et al., 2010). *I_N_* performed slightly better regarding smaller SNP sets and was selected as the basis for further SNP thinning.

In the first thinning method, we selected the SNP with the highest *I*_N_ score from each scaffold. This set thus comprises 10 SNPs, though only 6 of them were needed for 100% cross‐validated accuracy via a DAPC (Supporting Information Figure S5). For the second thinning method, SNPs with *R* ^2^ > 0.5 were grouped, resulting in 17 distinct clusters (Supporting Information Figure S4). We then selected the top‐ranking SNP from each cluster (again based on *I*_N_); from this reduced SNP set only 10 SNPs were needed for 100% cross‐validated accuracy via DAPC (Supporting Information Figure S5). When only the 33 transatlantic SNPs were used a small number of individuals were consistently miss‐assigned, indicating that these individuals could only be correctly assigned by adding information from the Northwest‐only SNPs (Supporting Information Figure S5). Nonetheless, 99.4% cross‐validated accuracy was achieved using just the six highest ranked (according to *I*_N_) transatlantic SNPs (Supporting Information Table S3). Overall, preliminary thinning decreased the number of SNPs needed for genetic characterization of spawning season (Supporting Information Figure S5).

### 3.3 Reproductive components in the BDO

We used the SNP panels to genetically assign spawning season in the BDO herring. Using all 64 SNPs, 84 individuals were assigned to the fall spawning component, and 13 to the spring spawning component. This was consistent with gonadal maturity: all nonspawning herring were assigned to the fall spawning component, and all but one ripe herring were assigned to the spring spawning component. Furthermore, the pattern of genetic clustering within the Bras d'Or sample appears remarkably similar to that of the GSL samples, with distinct fall and spring spawning clusters and a small number of genetically intermediate individuals (Supporting Information Figure S6); these genetically intermediate herring from both GSL and BDO showed notably high heterozygosity and were identified as F1, F2, and back‐crossed individuals by NewHybrid (Anderson & Thompson, 2002) (Supporting Information Figure S7).

**Figure 4:**
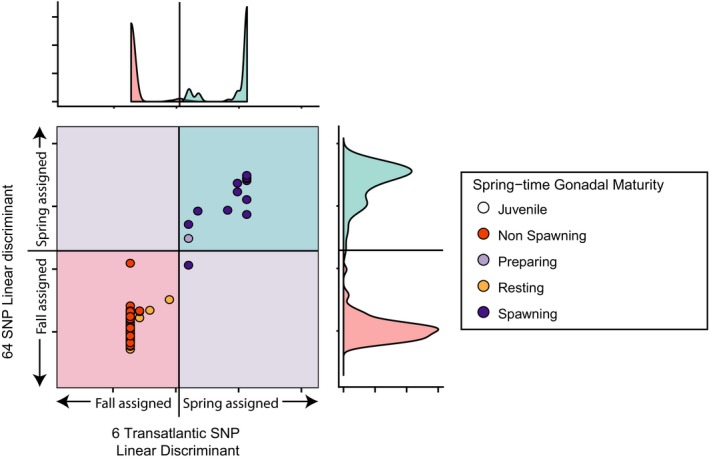
BDO herring (*N* = 97) of unknown spawning season distributed along two linear discriminants formed from the 6 most informative transatlantic SNPs and the linear discriminant created by all 64 SNPs. Both discriminants were created using the GSL samples (*N* = 148) shown in the density plots

Further DAPC based on SNP subsets all showed highly similar results (Supporting Information Figure S8). Using just 6 transatlantic SNPs, for example, divided BDO samples entirely according to gonadal maturity (Figure 4).

## 4 DISCUSSION

Using a panel of 64 highly informative SNPs, we corroborated that strong population structure exists within herring as a function of spawning season. These genomic regions of differentiation were proximate to a number of genes associated with reproduction by Lamichhaney et al. (2017)*,* and all but one SNP showed temporal stability between collections nearly a decade apart. The large effective population size of herring certainly plays a role in minimizing temporal change due to genetic drift, while emphasizing such local adaptation. Notably, the genomic differences here arose and persist despite a lack of physical barriers to gene flow. We also demonstrated how the link between these SNPs and spawning season can be applied in management; SNP selection based on both ranking and thinning methods can reduce the number of loci needed for accurate characterization of spawning season in herring. These SNP panels helped elucidate that Bras d'Or herring, previously considered to be exclusively spring spawning, comprise both of these differentially adapted spawning components; changes in the composition of Bras d'Or lake herring following stock collapse are likely to reflect changes in the relative abundance of these genetically differentiated spawning components, rather than phenotypic changes within a spawning component.

Biological differences between seasonal reproductive components, such as energy expenditure, fecundity, or spawning duration, have often been explained by plasticity (Brophy, Danilowicz, & King, 2006; Damme, Dickey‐Collas, Rijnsdorp, & Kjesbu, 2009; Gaggiotti et al., 2009; Petitgas, Secor, McQuinn, Huse, & Lo, 2010). However, the observed widespread genomic differentiation between these two spawning groups alongside previous research (Barrio et al., 2016; Bekkevold, Gross, Arula, Helyar, & Ojaveer, 2016; Lamichhaney et al., 2017) suggests that spawning season has a large genetic component in herring. These genomic regions may be related to biological responses to environmental differences intrinsic to spawning in different seasons, such as temperature or productivity. However, a number of these genomic regions are near genes with known roles in reproduction, implying that at least some differences are tied to the mechanisms controlling the timing of spawning. This appears to be a widespread phenomenon: factors influencing dispersal—which, in this case translates to those factors controlling spawning season fidelity—often co‐evolve with local adaptation to environmental heterogeneity (Kisdi, 2002).

Several SNPs were associated with genes of known function (see Lamichhaney et al., 2017), suggesting that this differentiation is indicative of adaptation. We observed that the locus of strongest differentiation between reproductive components was present on scaffold 1,420, which contains the gene encoding thyroid stimulating hormone (TSH) receptors, matching previous results (Lamichhaney et al., 2017). TSH is part of a conserved signaling pathway involving photoperiod‐controlled reproductive season in birds, mammals, and fish (Nakane & Yoshimura, 2014). In fact in salmonids, this pathway has been explicitly linked to gonadal maturity (Nakane et al., 2013).

Strong differentiation was also observed in SNPs upstream of the gene for calmodulin (CALM1), which is seasonally upregulated during spawning in goldfish (Zhang et al., 2009) and implicated in light‐controlled responses in the pineal gland (Bustos et al., 2011). Similarly, differentiation appears at SNPs within introns of estrogen receptor gene 2a (ESR2a), a protein essential for reproduction in zebrafish (Lu, Cui, Jiang, & Ge, 2017). However, a large number of these genes had no known role in reproduction. The only SNP to show significant temporal variation was proximate to the TTC3 gene, which is relatively unstudied beyond human applications.

Both recruitment and abundance of herring in the Northwest Atlantic were higher prior to 2005(DFO, 2016a). If seasonal straying is density‐dependent (McQuinn, 1997b) we see no indication that this dilutes the observed divergent adaptation. Certainly, it appears that often reproductive components in herring remain distinct over time, both demographically (Larsson, Laikre, André, Dahlgren, & Ryman, 2010; Stephenson et al., 2009) and, as shown in this study, in terms of their adaptive genetic variation. Still, straying between spawning seasons likely occurs at low and steady rates, consistent with observations in the Gulf of St Lawrence (Graham, 1962; McQuinn, 1997b) and the Northeast Atlantic (Brophy et al., 2006). Indeed, a small number of hybrids were identified (see Supporting Information Figure S7); notably, the majority of these were back‐crossed individuals. Selection against migrants (and the resultant hybrids) may help maintain the observed local adaptation (McQuinn, 1997a; Spichtig & Kawecki, 2004) and explain the apparent lack of F1 and F2 hybrids. Intermediate migration patterns, often observed in hybrids (Moore et al., 2010; Pujolar et al., 2014), may also result in underrepresentation of hybrids in our samples. Overall, Atlantic herring appears to be a striking example of persistent local adaptation alongside seasonal differences in spawning migrations, despite a lack of physical barriers. This contrasts with evidence of adaptation in cod that is either temporally unstable (Poulsen et al., 2011; Therkildsen, Hemmer‐Hansen, Als, et al., 2013; Therkildsen, Hemmer‐Hansen, Hedeholm, et al., 2013) or if temporally stable, it is linked to chromosomal rearrangements (e.g., inversions) as seen in the differentiation between migratory and stationary cod ecotypes (Berg et al., 2016; Kirubakaran et al., 2016) shown to be present on both sides of the Atlantic Ocean (Berg et al., 2017).

We found substantial redundancy in these 64 SNPs; we obtained similar results regardless of the metric used to rank SNP usefulness, likely a consequence of using a highly informative subset (Ding et al., 2011; Storer et al., 2012; Wilkinson et al., 2011). Still, our analysis, like others (Ding et al., 2011), found *I*_N_ to be the best performing metric. Further SNP thinning also proved to decrease the number of SNPs needed for accurate characterization of spawning season to as low as six SNPs. Hierarchical clustering continues to be an effective method of marker thinning (Cho & Dupuis, 2009; Rinaldo et al., 2005), while thinning by genomic region or SNP range produced similar results.

The Bras d'Or Lake (BDO) is the largest inland sea in North America, unique in both ecology and physical geology (Yang, Sheng, Hatcher, & Petrie, 2007). Historically, spring spawning has been prevalent within BDO (Denny, Clark, Power, & Stephenson, 1998). However, genotyping a small sample of BDO herring uncovered both spring and fall spawning components coexisting in the lake. The latter group included the majority of samples collected in BDO in 2016%, and 100% of 2017 individuals, suggesting that genetically‐distinct fall spawners may now be widespread in BDO, though the sample sizes and geographic coverage in the present study are not appropriate for an assessment of relative abundances. Yet a change in abundance of fall and spring spawning components in BDO taking place alongside the collapse of the spring spawning population due to overfishing between 1996 and 2000 is certainly possible. Indeed, fall spawning herring within the Bras d'Or lake were first reported in 1996, coupled with decreasing spring landings (Stephenson et al., 1999). Anecdotal evidence from this time period includes uncharacteristic October migrations and “black‐back” herring (characteristic of the Scotian Shelf [Crawford, 1979]) in BDO following stock collapse (Denny et al., 1998). The pattern of genetic clustering of the components found within BDO appears to match that of the GSL herring and the observed temporal stability in the GSL suggests a genetic shift from within the spring spawning component is unlikely. Certainly, the genetic differences between spawning components strongly supports the hypothesis that if there are currently more fall spawning herring than in the past, this is because of changes in the components' relative abundance rather than because of a phenotypic change within component. This newly described BDO fall spawning component likely marks the success of a previously undetected (and possibly oceanic) fall spawning component. Notably, adaptive and ecological divergence between subpopulations, such as the observed differences between spawning components, suggests they may not occupy the same niche (Crandall, Bininda‐Emonds, Mace, & Wayne, 2000), although the implications of this in herring remains unknown. Nevertheless, the fall spawning component may have partially replaced the spring spawning component, dampening the overall decrease in herring abundance within BDO. This may be a remarkable demonstration of the portfolio effect (Schindler et al., 2010). More intensive and widespread sampling within the BDO Lake is necessary to fully answer these questions, however.

The reference 64 SNP panel has shown temporally (this study) and spatially (Lamichhaney et al., 2017) robust differentiation. We have demonstrated that genetic characterization of spawning season can now be done with a reduced number of SNPs, as verified using cross‐validation methods. It is both important and now realistic that any assessment of the genetic composition of herring includes the genetics of spawning season. The unique and temporally stable divergence of seasonal components should inform management decisions in the Northwest Atlantic. Certainly, precautionary management should take into account differences in adaptive genetic variation and life‐history traits and their implications in subpopulation recovery and the portfolio effect, while aiming for the preservation of both fall and spring spawning components.

## CONFLICT OF INTERESTS

The authors declare no competing financial interests.

## AUTHOR CONTRIBUTIONS

D.E.R. and A.P.F.P. designed the study; Q.K., J.K. A.P.F.P and J. L. M. performed tissue collection, laboratory work and data analysis; Q.K. wrote a first draft of the paper and all authors contributed to the writing of the final document. All authors approved the manuscript before submission.

## DATA ACCESSIBILITY

The raw genotyped SNP data used in this study will be made available in a public repository upon acceptance.
