# Supplementary material for: Temporal stability and assignment power of adaptively divergent genomic regions between herring (Clupea harengus) seasonal spawning aggregations
Source: Ecol Evol. 2018 Dec 11;9(1):500–10. doi: 10.1002/ece3.4768 (PMC6342187; doi:10.1002/ece3.4768)
Supplement: Supplementary file 2 [file ECE3-9-500-s002.docx]

**Supplemental Information for:**

**Stability and assignment power of genomic differences between herring seasonal spawning aggregations**

Quentin Kerr^1^, Angela P. Fuentes-Pardo^1^, James Kho^1^, Jenni L. McDermid^2^, Daniel E. Ruzzante^1^

**Table of Contents:**

| **Figure S1** | Page 2 |
| --- | --- |
| **Figure S2** | Page 2 |
| **Figure S3** | Page 3 |
| **Figure S4** | Page 4 |
| **Figure S5** | Page 5 |
| **Figure S6** | Page 6 |
| **Figure S7** | Page 7 |
| **Figure S8** | Page 8 |
| **Table S1** | Page 9 |
| **Table S2** | Page 10 |

# **Supplemental Figures**


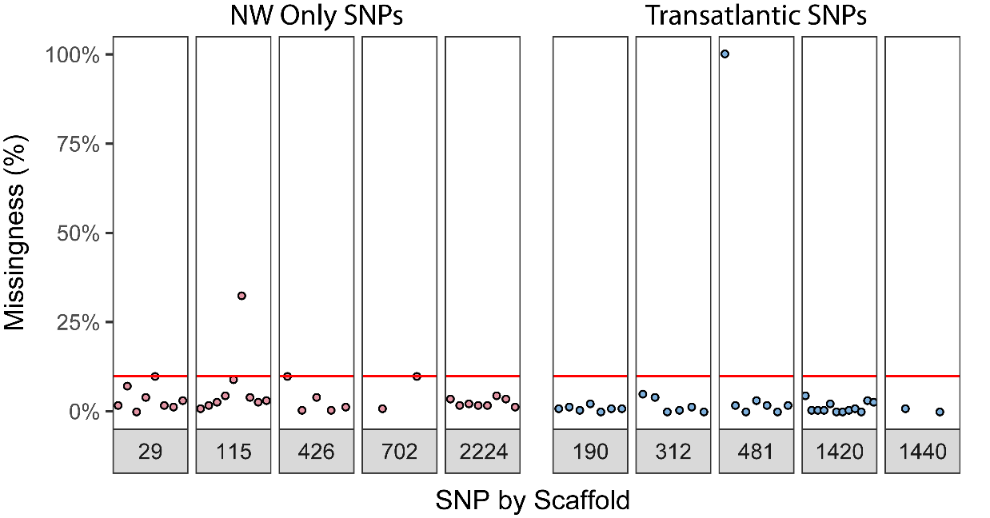


Figure S1. The percentage of unsuccessfully genotyped individuals per SNP, out of 276 attempted herring samples. Two SNPs with over 10% missing data were removed: CLUHAR0004 and CLUHAR00111.


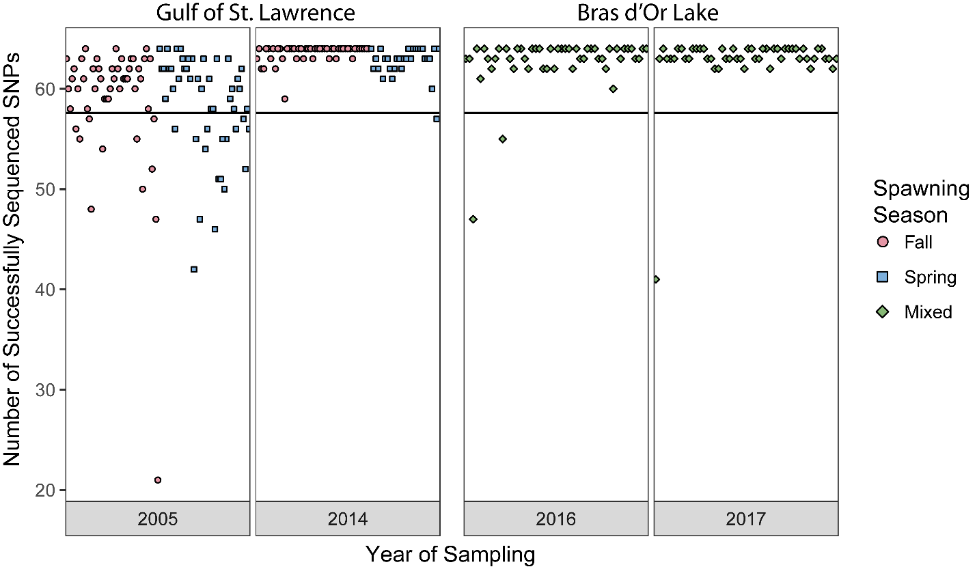


Figure S2. The number of SNPs successfully amplified for each individual herring sample, showing some increased failure in older samples. 31 individuals were removed as they were successfully genotyped at fewer than 90% of the SNPs (i.e. <58 SNPs).


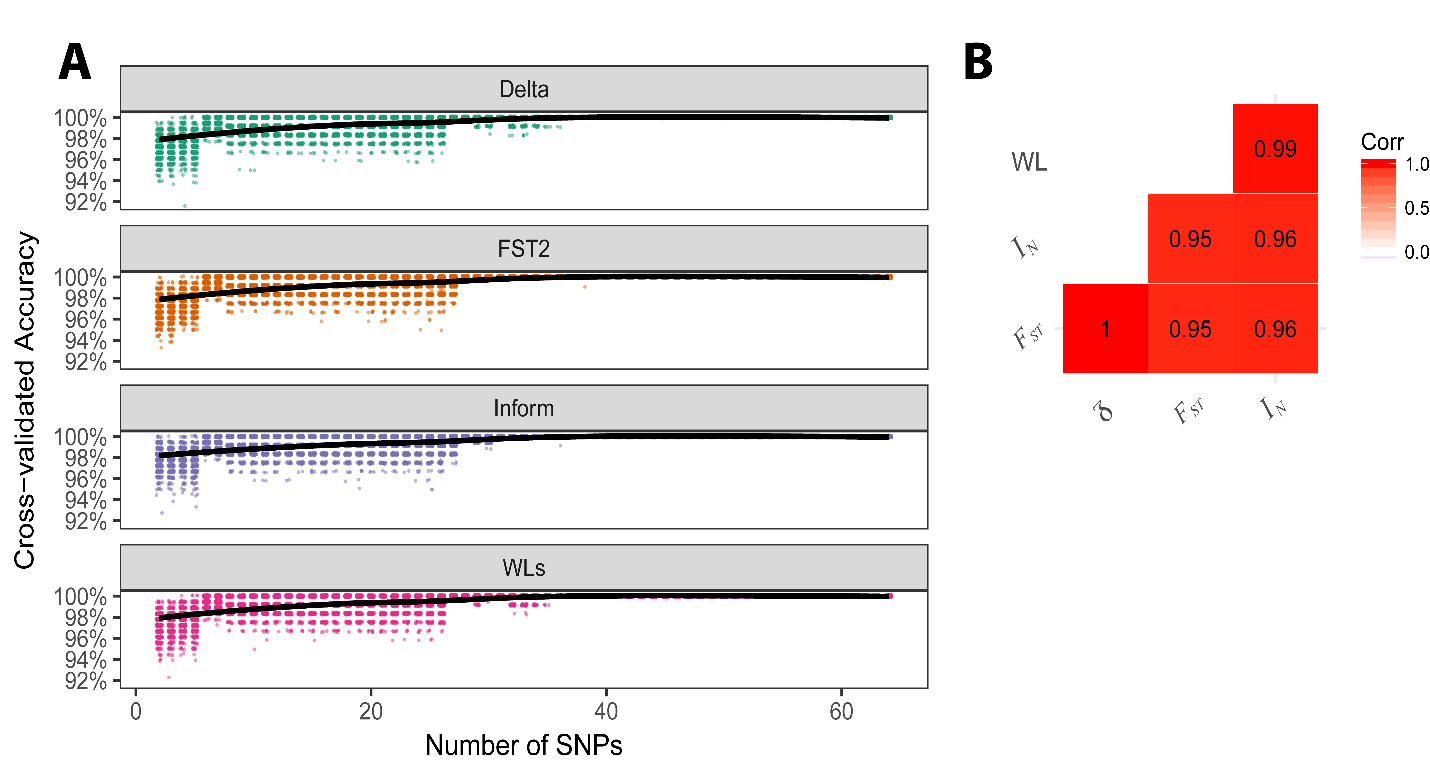


Figure S3. Cross-validated accuracy of DAPCs based on increasingly large sets of SNPs as ranked by δ, *F_ST_*, *I_n_*, and *WL* scores (**A**). Cross-validated accuracy was obtained via the GSL herring samples (N=148) and uses 300 repetitions; data was divided into 90% training set, and 10% testing set. Correlation (R^2^) of these metric estimates regarding the 64 SNPs, based on GSL samples, is also shown (**B**).


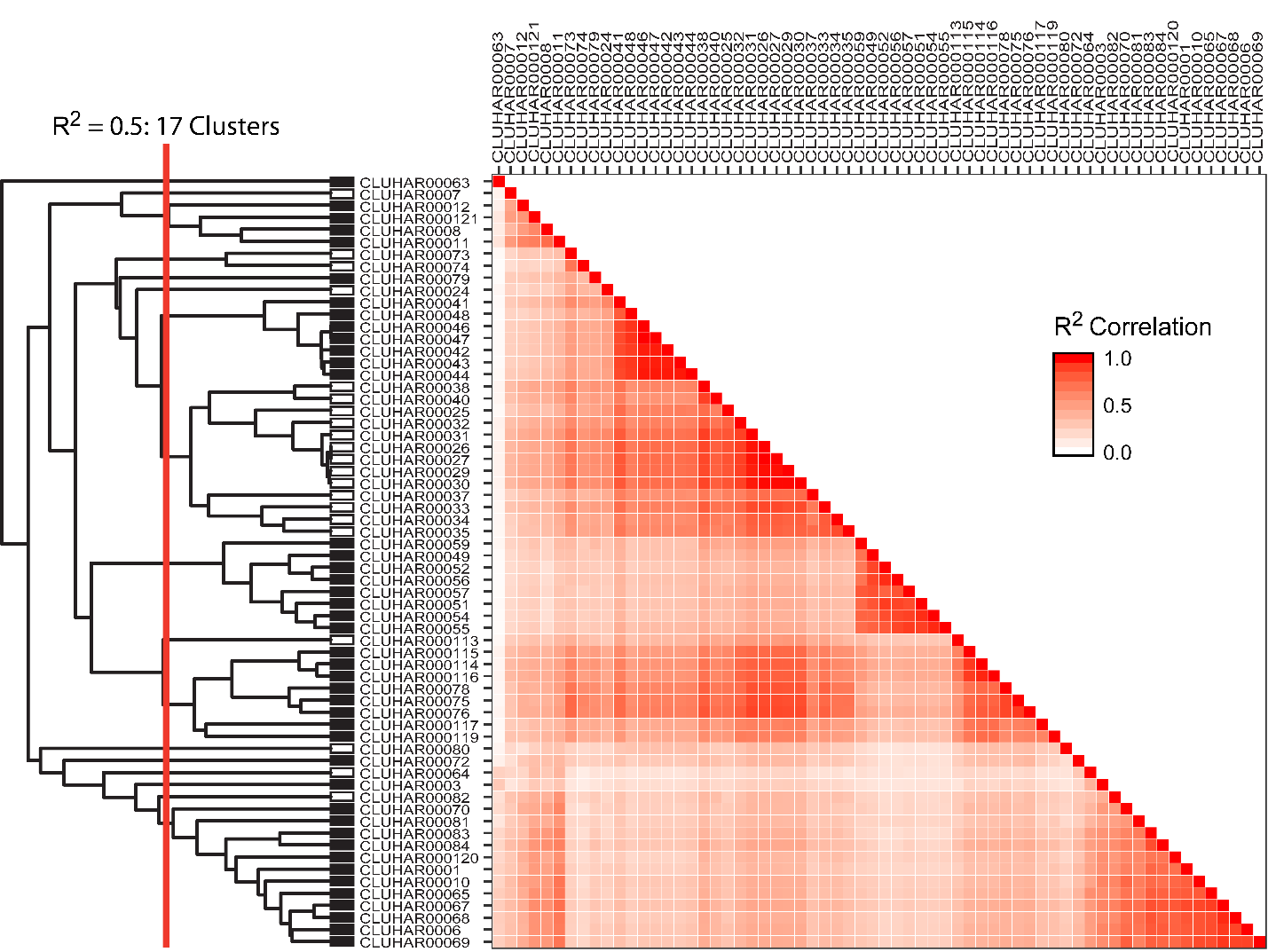


Figure S4. SNP correlation (R^2^) and subsequent hierarchical complete-linkage clustering resulting in 17 clusters (denoted with boxes) with a cut-off of R^2^ = 0.5; the top-ranked SNP from each cluster was retained for the non-clustered thinning method.


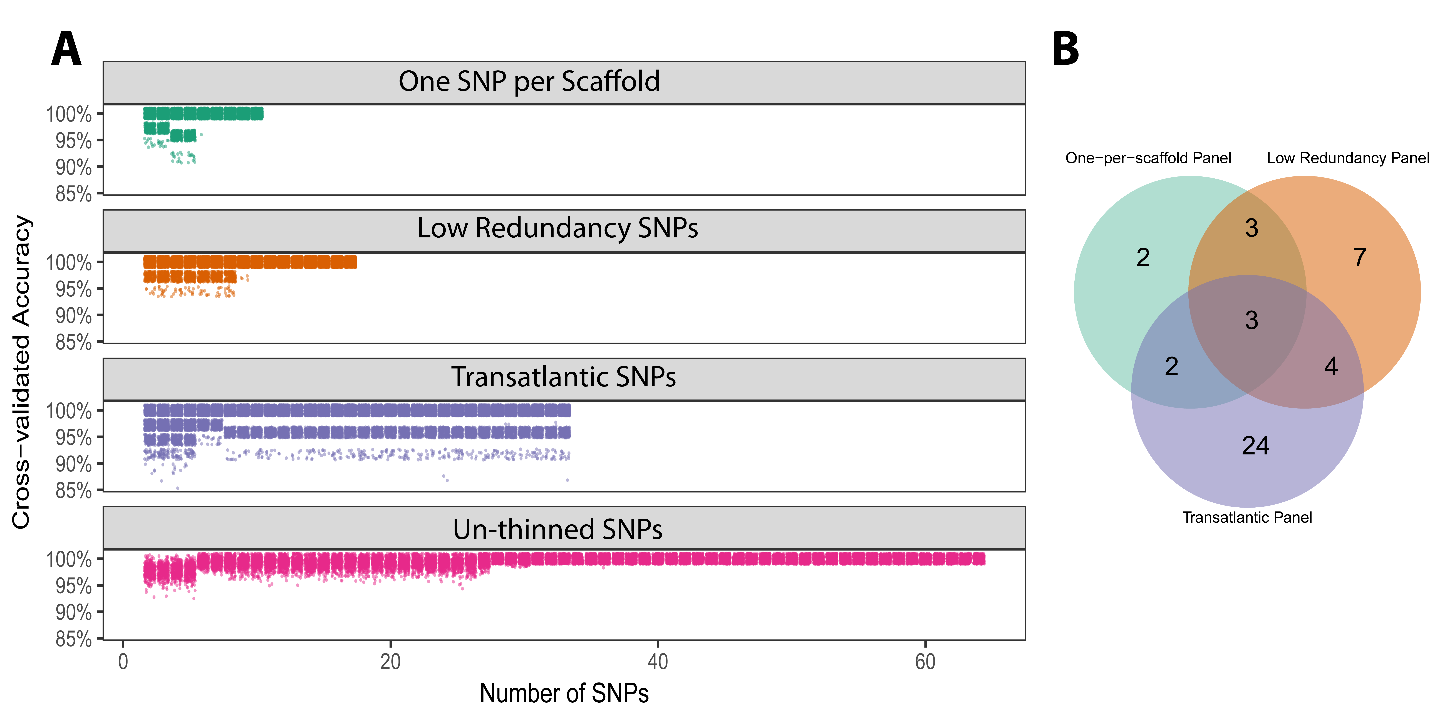


Figure S5. Cross-validated accuracy of DAPCs based on increasingly large sets of SNPs, as ranked by *I_N_*, and thinned via three methods: physically-unlinked SNPs were limited to 1 per scaffold, non-clustered SNPs were limited to 1 per linkage-disequilibrium cluster, and transatlantic SNPs were limited to those found on both sides of the Atlantic (**A**). Cross-validated accuracy was obtained via the GSL herring samples (N=148) and uses 1000 repetitions of 10% resampling. The amount of overlap between thinning methods is also shown.


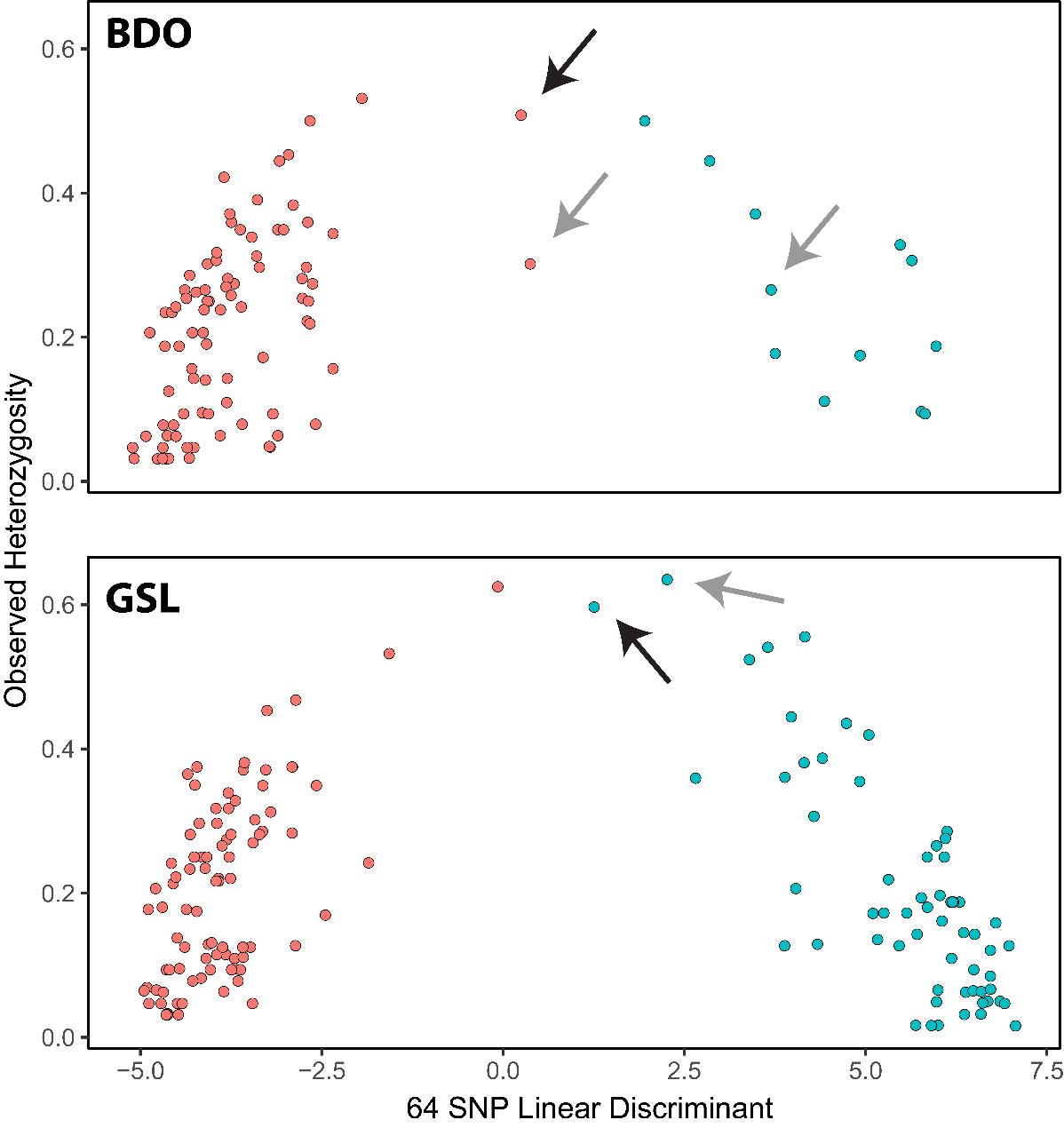


Figure S6. The proportional heterozygosity of individual herring genotyped at 64 SNPs in the BDO Lake (N=97) and the GSL (N=148) compared with their distribution along the 64 SNP linear discriminant. Individuals that appear to be genetically intermediate in terms of the linear discriminant also appear to have the highest proportion of heterozygosity; individuals are identified by NewHybrid as likely F1 (black arrows) and F2 (grey arrows) hybrids.


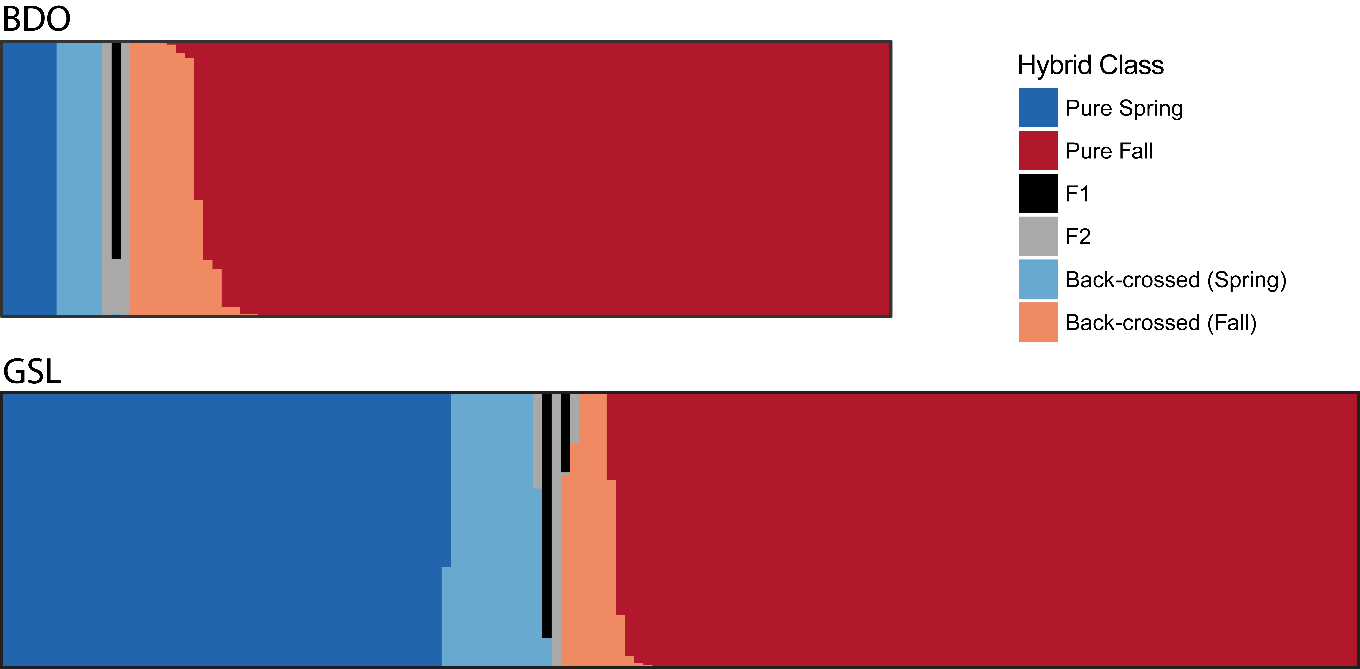


Figure S7. NewHybrid Posterior probability plot for N=97 Atlantic herring from BDO (N=97) and (N=148) herring from GSL; each bar is an individual, and the probability that this individual belongs to a particular hybrid class is given by the proportion of each color.


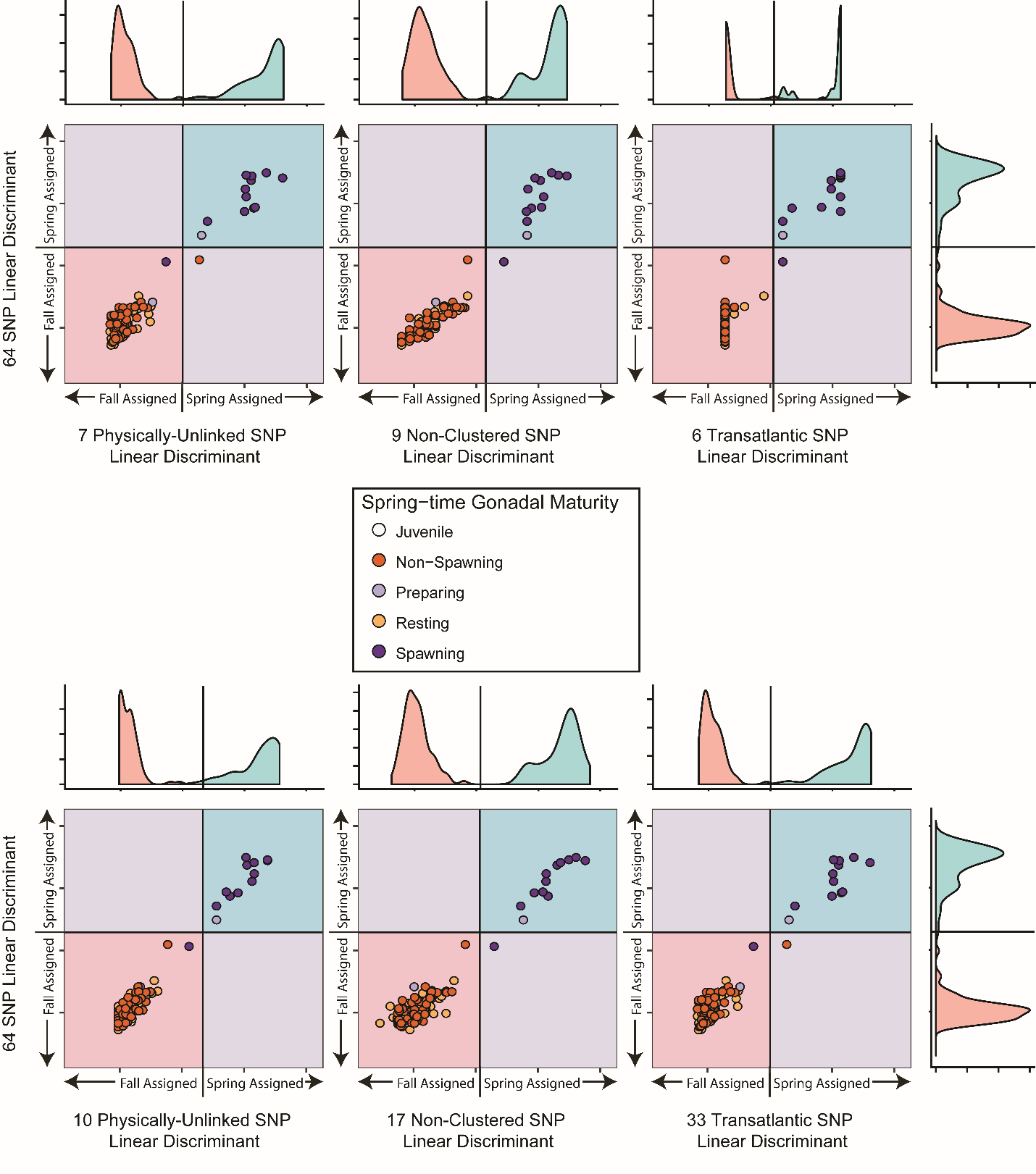


Figure S8. Comparison of the distributions of an unknown spawning sample from BDO along linear discriminants resulting from screened and ranked SNP subsets against the full 64-SNP linear discriminant; the GSL samples were used to create each linear discriminant, and are shown via density plots, separated into fall-spawning (red) and spring-spawning (blue). Gonadal maturity of BDO samples at the time of catch (spring) is shown. Note that individuals that fall within the purple squares (top-left and bottom right) were not assigned to the same component by both methods.

Table S1. Samples used, including locations and sample specifics; N is the original dataset, and N’ refers to individuals genotyped at >90% of loci. (N = 276; N’ = 245)

|  | | | | | | |
| --- | --- | --- | --- | --- | --- | --- |
| **Sample Location** | **NAFO Division** | **Year** | **Catch season** | **Gonadal Maturity at time of catch** | **N (N’)** | **Date of Sampling** |
| Gulf of St. Lawrence | 4TH | 2005 | Spring | Spawning | 48 (32) | Apr. 28 - May 9 |
| Gulf of St. Lawrence | 4TL | 2005 | Fall | Spawning | 49 (38) | Aug. 31 - Sep. 6 |
| Gulf of St. Lawrence† | 4TH | 2014 | Spring | Spawning | 30 (29) | May 1 - Apr. 30 |
| Gulf of St. Lawrence | 4TL | 2014 | Fall | Spawning | 49 (49) | 25-Aug |
| Bras d'Or Lakes | 4VWX | 2016 | Spring | Mixed | 50 (48) | 28-Apr |
| Bras d'Or Lakes | 4VWX | 2017 | Spring | Unknown | 50 (49) | 20-May |
| † Samples sequenced in Lamichhaney *et al.* 2017 | | | | |  |  |

Table S4. Polymerase Chain Reaction specifications including both concentrations and cycle temperatures.

| PCR concentrations |
| --- |
| 4.5 μL of dd·H2O |
| 1 μL of 10× reaction buffer (Bio Basic Inc., Markham, Ontario) |
| 2.0 mM of MgSO4 (Bio Basic Inc., Markham, Ontario) |
| 0.1 μM of forward primer |
| 0.1 μM of reverse primer |
| 200 μM dNTPs (Bio Basic Inc., Markham Ontario) |
| 0.25 U TSG Polymerase (Biobasic Inc., Markham, Ontario) |
| 2.0 uL of genomic DNA |
| Total: 10 μL Mix |
|  |
| PCR Cycle Program (6 μl volume reaction) |
| 1 cycle: 5 min 94◦C (initial denaturation) |
| 45 cycles: 20 sec 94◦C (denaturation) |
| 30 sec 56◦C (annealing) |
| 1 min 72◦C (extension) |
| 1 cycle: 3 min 72◦C (final extension) |
| Final step: indefinite 4◦C (hold) |
| Total: ~2.5 hours |
